# Clone wars: From molecules to cell competition in intestinal stem cell homeostasis and disease

**DOI:** 10.1038/s12276-022-00854-5

**Published:** 2022-09-18

**Authors:** Gabriele Colozza, So-Yeon Park, Bon-Kyoung Koo

**Affiliations:** 1 Institute of Molecular Biotechnology of the Austrian Academy of Sciences (IMBA), Vienna Biocenter (VBC), Vienna, 1030 Austria; 2 Center for Genome Engineering, Institute for Basic Science, 55, Expo-ro, Yuseong-gu, Daejeon, 34126 Republic of Korea

**Keywords:** Intestinal stem cells, Colorectal cancer

## Abstract

The small intestine is among the fastest self-renewing tissues in adult mammals. This rapid turnover is fueled by the intestinal stem cells residing in the intestinal crypt. Wnt signaling plays a pivotal role in regulating intestinal stem cell renewal and differentiation, and the dysregulation of this pathway leads to cancer formation. Several studies demonstrate that intestinal stem cells follow neutral drift dynamics, as they divide symmetrically to generate other equipotent stem cells. Competition for niche space and extrinsic signals in the intestinal crypt is the governing mechanism that regulates stemness versus cell differentiation, but the underlying molecular mechanisms are still poorly understood, and it is not yet clear how this process changes during disease. In this review, we highlight the mechanisms that regulate stem cell homeostasis in the small intestine, focusing on Wnt signaling and its regulation by RNF43 and ZNRF3, key inhibitors of the Wnt pathway. Furthermore, we summarize the evidence supporting the current model of intestinal stem cell regulation, highlighting the principles of neutral drift at the basis of intestinal stem cell homeostasis. Finally, we discuss recent studies showing how cancer cells bypass this mechanism to gain a competitive advantage against neighboring normal cells.

## Cancer: Tumors gain competitive edge over intestinal stem cells

Stem cells in the gut rapidly renew themselves through processes that cancer cells co-opt to trigger tumor development. Gabriele Colozza from the Institute of Molecular Biotechnology in Vienna, Austria, and colleagues review how a network of critical molecular signals and competition for limited space help to regulate the dynamics of stem cells in the intestines. The correct balance between self-renewal and differentiation is tightly controlled by the so-called Wnt signaling pathway and its inhibitors. Competition between dividing cells in the intestinal crypts, the locations between finger-like protrusions in the gut where stem cells are found, provides another protective mechanism against runaway stem cell growth. However, intestinal cancer cells, thanks to their activating mutations, bypass these safeguards to gain a survival advantage. Drugs that target these ‘super-competitive’ behaviors could therefore help combat tumor proliferation.

## Introduction to the intestinal epithelium: A “conveyor belt” of different cell types

Many of our organs and tissues require constant cellular turnover throughout life to remain functional. This continuous self-renewal is fueled by dedicated multipotent stem cells, which have a life-long ability to divide and generate the different cell types that comprise specific adult tissues. In mammals, the small intestine is the fastest self-renewing tissue, with its entire epithelium being completely replenished every 3–5 days^1,2^, thus representing an ideal system for studying the biology of adult stem cells and their specialized niche. Because of the harsh conditions of the intestinal lumen, millions of cells die every day and are rapidly replaced by newly generated differentiated cells originating from actively dividing stem cells in the crypt^1^. This life-long renewal ability allows the intestinal epithelium to retain its digestive and absorptive functions and to maintain a barrier against infectious microorganisms or toxic substances without being severely compromised.

**Fig. 1:**
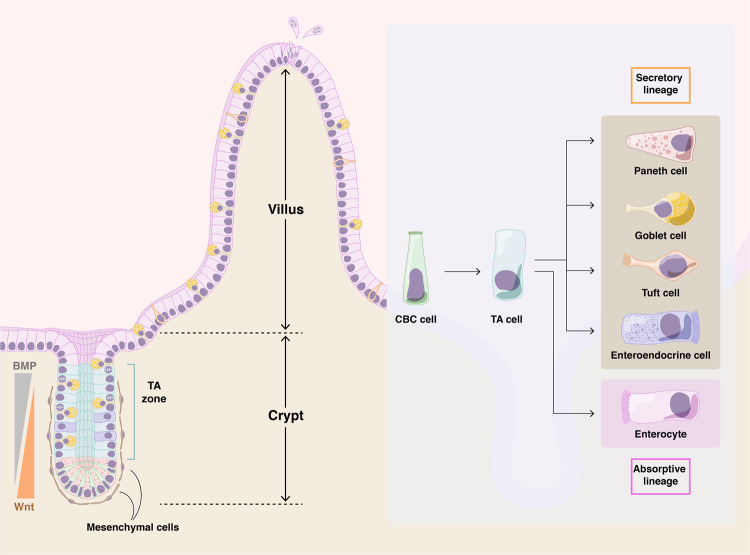
Organization of the intestinal villus and crypt. Crypts of Lieberkühn surround intestinal villi (six or more crypts per villus) and replenish them with newly formed cells. Crypt base columnar (CBC) stem cells, located at the base of each crypt, represent the driving force that maintains intestine homeostatic renewal. CBC cells divide and originate transit-amplifying (TA) cells located in the upper zone of the crypt, which undergo multiple rounds of mitosis before differentiating into any of the cells of either secretory or absorptive lineages (right panel). CBC cells are intermingled with Paneth cells, specialized secretory cells that support CBC cells by providing growth factors. The balance between self-renewal and differentiation is regulated by morphogenetic signals, including BMP and Wnt. Differentiated cells move to the top of the villus, where they are shed into the intestinal lumen and die by anoikis at the end of their life cycle.

Cells of the small intestine are organized into a simple columnar epithelium that folds into long finger-like structures called villi and projects into the intestinal lumen (Fig. 1). In mice, this basic architecture is established between embryonic Day E13.5 and E18.5, when the pseudostratified gut endoderm transitions into the epithelial monolayer^3,4^. Once matured postnatally, functional compartmentalization is completed: specialized cells are located in the villi, while proliferating progenitor and stem cells occupy the crypts of Lieberkühn (hereafter simply referred to as ‘crypts’)^5,6^. Intestinal stem cells (ISCs) reside at the base of each crypt and are also known as crypt base columnar (CBC) cells. These stem cells are readily distinguishable by their slender, wedge-like appearance and scant cytoplasm. Upon cell division, CBC cells produce highly proliferative short-lived progenitors known as transit amplifying (TA) cells, which are located higher in the crypt. TA cells undergo multiple rounds of rapid cell division while differentiating into several cell types^7^. Differentiating progenitor cells migrate upward along the crypt-to-villus axis (similar to a cellular “conveyor belt”) to produce terminally differentiated cell types in the villus (Fig. 1): enterocytes (belonging to the absorptive lineage), whose function is to complete the breakdown of partly digested food and absorb nutrients; and cells of the secretory lineage, which include mucus-secreting goblet cells; enteroendocrine cells, which produce hormones; Paneth cells, which secrete antimicrobial agents, such as cryptdins and lysozyme; and other minor cell types (Tuft cells and Peyer’s patch-associated M-cells). Paneth cells, unlike the other cell types, migrate downward toward the crypt base, where they intercalate with the CBC stem cells. Upon reaching the tip of the villus, cells ultimately die by *anoikis*, an anchorage-dependent form of cell death^7,8^, and are shed into the gut lumen.

## Intestinal stem cells: From homeostasis to regeneration

As noted above, the bases of intestinal crypts harbor actively dividing CBC stem cells. During development, CBC cells derive from the proliferating cells that populate the intervillus regions, although the high level of plasticity allows even nonproliferating cells of the nascent villi to contribute to the adult intestinal stem cells, in part due to the intense remodeling of the fetal intestinal architecture^9^. CBC cells divide at least once every 24 h and were described as potential intestinal stem cells several decades ago based on fate-mapping experiments using tritiated [^3^H]thymidine^10,11^. The formal confirmation that CBC cells are indeed the actual adult intestinal stem cells only came more recently, when specific genetic markers were used for genetic lineage tracing (see below). One of these markers is Lgr5 (Leucin-rich repeat containing G-protein coupled receptor 5), which is specifically expressed in the crypt base, coinciding with CBC cells^12^.

The identification of stem cell-specific markers opened the possibility for elegant lineage tracing experiments. Lineage tracing is a fate-mapping tool^13^ that involves introducing a genetically encoded, heritable mark into a putative stem cell. This mark is inherited by the progeny of initially labeled stem cells, allowing their tracking and characterization. Using the *Lgr5-eGFP-IRES-CreERT2;R26R-LacZ* knock-in mouse model, it was possible to visualize not only the CBC stem cells with green fluorescence but also their direct descendants with β-galactosidase (LacZ) expression triggered by the stem cell-specific activation of tamoxifen-inducible CreERT2 recombinase: ribbons of cells emanating from the crypt bottoms were observed running along the sides of the crypts and comprised the entire spectrum of differentiated intestinal epithelial cells^12^. Furthermore, labeled cells were still produced over long periods of time, indicating the self-renewal ability of the initially labeled CBC cells. Thus, Lgr5^+^ CBC cells meet the essential requirements to be considered tissue-resident adult stem cells: multipotency and long-term self-renewal. In addition to *Lgr5*, other intestinal stem cell-specific markers have been identified, including *olfactomedin 4* (*Olfm4*), *Troy* (also known as *Tnfrsf19*), *Ascl2*, *Smoc2*, *Rnf43,* and *Znrf3*^14–20^.

Further proof of CBC stemness potential came from ex vivo culture experiments on Lgr5-eGFP reporter mice using single Lgr5^+^ cells isolated by fluorescence-activated cell sorting (FACS). The isolated cells could generate 3D self-renewing and self-organizing epithelial structures, denoted as ‘organoids’, which recapitulated the architecture and cellular composition of the adult intestine^21^. Intestinal organoids can form in the absence of mesenchymal cells and require a limited set of growth factors, namely, EGF, Noggin, and R-spondin^21^. Of note, Lgr5^+^ stem cells (or cells expressing Lgr6, another paralog) were identified in several other tissues, including the colon, hair follicle, stomach pylorus and corpus, mammary glands, ovarian surface epithelium, and more recently, skeletal muscle^12,22–27^. The adult stem cell and organoid research fields have developed considerably after the initial success of mouse intestinal organoid culture, holding great promise as novel in vitro models for studying organogenesis and organ maintenance as well as related human diseases^28–32^.

In addition to crypt base stem cells, for a long time, it has been speculated that alternative intestinal stem cells could reside in position +4 (with respect to the crypt base), where rare DNA label-retaining cells (LRC) were found^33–36^. DNA label retention is a feature of slowly cycling cells, which remain in a quiescent state^37^. These cells are thought to be different from Lgr5^+^ stem cells, as they do not actively divide in homeostatic conditions but re-enter the cell cycle following tissue damage that depletes Lgr5^+^ cells, driving the regenerative process that reconstitutes the injured tissue. Several genetic markers have been reported as enriched in position +4, such as *Bmi1*^38,39^, *Tert*^40^, *Hopx*^41^, and *Lrig1*^42^, although the specificity of these markers has been debated^16^, as described below. Using diphteria toxin receptor (DTR) mouse genetic models that allow the selective ablation of CBC cells, non-Lgr5^+^ progenitor cells were shown to act as a reserve stem cell pool that could regenerate all intestinal cell types, including Lgr5^+^ stem cells^43^. Notably, the intestinal architecture was maintained even after prolonged damage, suggesting that Lgr5^+^ cells are in theory dispensable for tissue maintenance. However, the specificity of the +4 markers has been questioned after the finding that Lgr5^+^ crypt base stem cells also express robust levels of these genes^16^. In-depth analysis of the Lgr5^+^ cell transcriptome and proteome provided a combined stem cell signature of approximately 500 genes and proteins enriched in Lgr5^+^ ISCs, which included putative +4 markers^16^. Single-molecule fluorescence in situ revealed that +4 markers are not confined to a distinct stem cell population; rather, they show broad expression profiles throughout the crypt that overlap with Lgr5^+^ stem cells^44^. More recently, single-cell RNA sequencing (scRNA-seq) analyses have led to similar conclusions, showing that several of these genes, including *Bmi1*, are expressed in multiple cell clusters at similar levels^45,46^. Taken together, these findings suggest the presence of progenitor cell populations with strong plasticity, but the identification of their marker genes has not been fully established.

Several other studies have highlighted the shared plasticity among differentiating progenitors both in secretory and absorptive lineages in the intestinal epithelium. Within the secretory lineage, Delta-like 1^+^ (Dll1^+^) cells give rise to all secretory cell types (enteroendocrine, tuft, goblet, and Paneth) during homeostasis^47^, indicating that these rare Dll1+ cells are indeed secretory progenitors. Lineage tracing experiments coupled with irradiation to kill actively cycling Lgr5^+^ cells showed that Dll1^+^ secretory progenitors can revert to Lgr5^+^ cells and form long-lived clones along the villus axis, consisting of both absorptive and secretory cells^47^. Similar observations were also reported by using an elegant genetic labeling strategy to mark the LRCs in the crypt. This work demonstrated that the LRCs in the crypt are normally secretory progenitors, which can show a broader clonogenic potential by dedifferentiating and regaining multipotent stem cell features after tissue injury^48^. In addition, using the Alpi-IRES-CreERT2 knock-in allele, early enterocyte progenitors were also found to have plasticity comparable to that of their secretory counterparts when Lgr5+ stem cells were specifically depleted^49^. Due to their abundance, it seems that enterocyte progenitors are able to form a larger reservoir of stem cells during crypt regeneration. However, enterocyte progenitors are also actively cycling, so irradiation would deplete them as well as Lgr5+ stem cells, leaving the slow-cycling secretory progenitors to hold a key role in tissue regeneration. Therefore, depending on the types of injury, the two lineage progenitors will be actively and selectively adopted for the re-establishment of the intestinal stem cell pool.

Interestingly, achaete-scute-like 2 (Ascl2) has been recently identified as a key factor for the process of intestinal epithelial cell dedifferentiation^45^. Ascl2 is a basic helix-loop-helix transcription factor whose expression is under Wnt regulation in the intestinal crypt and is required for stem cell survival and identity^15,20^. Upon treatment with tamoxifen, ISC descendants of *Lgr5*^*GFP-CreER(T2)*^; *R26R*^*tdTom*^ mice were permanently labeled with a red fluorescent marker (tdTomato). After crypt stem cells were eliminated through radiation, intestinal crypts were soon repopulated by stem cells expressing not only GFP (under the control of Lgr5 promoter) but also tdTomato, indicating that these new stem cells originated from their recent progeny, e.g., cells that were on their way to differentiation^45^. Ascl2 was ectopically expressed in cells well above position +5 and was absolutely required for the recovery of ISCs upon damage, probably by the activation of a regenerative transcriptional program including interleukin receptor *Il11ra1* among its targets. Additionally, this study showed that not only secretory progenitors but also absorptive lineages were able to recover lost ISCs, as observed in earlier work^49^.

More recently, an injury-responsive stem cell population has been identified, expressing high levels of the stress-response gene Clusterin (Clu^+^)^46^. These Clu^+^ cells show characteristics of a fetal gene signature, and their appearance depends on active Yap1, a transcription factor inhibited by Hippo signaling. Under different types of damage, Clu^+^ cells were able to repopulate CBC-depleted crypts, reconstituting all the main intestinal cells, including CBC cells, in a hierarchical manner^46^. Nevertheless, whether Clu+ cells derive from preexisting Clu+ cells or from ISCs and other progenitors through reprogramming in response to injury remains ambiguous. Notably, Yap has been found to induce the reprogramming of colonic epithelial and stem cells following lesions, generating a population of Klf6^+^ cells characterized by a wound-healing gene signature and low Wnt activity^50^. While several works have shown that Yap is required for intestinal regeneration^50–52^, it is unclear whether all the cells participating in regeneration, including the abovementioned absorptive and secretory lineages, undergo Yap-driven reprogramming.

In conclusion, Lgr5^+^ CBC cells represent the workhorse that sustains the life-long homeostatic self-renewal of the intestine. On the other hand, after epithelial tissue damage, various cell types participate in the regeneration process depending on the contexts and available populations that remain intact in the tissue (see Table 1). How all these identified cell types are orchestrated to perform efficient tissue repair is incompletely characterized, but the players and signaling pathways involved are being rapidly revealed, leading to a clearer understating of not only normal regeneration but also chronic tissue damage.

**Table 1:** Summary of intestinal stem cell marker genes and properties.

ISC marker	Functions and characteristics	Reference
CBC cells		
Lgr5	Lgr5+ CBC cells sustain the life-long homeostatic self-renewal of the intestine.	^12^
Troy	Troy+ cells are capable of the long-term renewal of the epithelium and correspond to Lgr5+ CBC cells. Troy suppresses signaling mediated by R-spondin and Wnt agonists.	^18^
Olfm4	Olfm4+ cells generate all major intestinal epithelial cell types. Olfm4 is robustly and specifically expressed in CBC cells, although its function is not yet fully elucidated.	^14,19^
Ascl2	Ascl2 is crucial for the maintenance of Lgr5+ CBC cells by binding to promoters and enhancers of its target genes. Ascl2, together with β-catenin and Tcf4, synergistically activates the genes fundamental to the stem cell state.	^15,20,45^
Smoc2	Smoc2 expression is restricted to CBC cells, and Smoc2+ cells show the typical stem cell tracing signature of Lgr5+ cells.	^16^
Rnf43/Znrf3	Rnf43 and Znrf3 regulate the homeostasis of CBC cells by acting as fundamental feedback inhibitors of Wnt signaling.	^17,102^
Alternative ISCs that reside in position +4		
Bmi^a^	Bmi+ cells are usually quiescent and serve as injury-inducible reserve ISCs that generate Lgr5+ CBC cells and all intestinal epithelial cell types. Bmi+ cells are insensitive to Wnt signaling blockade or activation, while Lgr5+ cells are sensitive.	^38,39^
Tert^a^	Tert+ cells are usually quiescent and relatively resistant to irradiation-induced cell death. Tert+ cells give rise to Lgr5+ CBC cells and contribute to the regeneration of intestinal epithelium following injury.	^40^
Hopx^a^	Hopx+ cells are slow-cycling ISCs that give rise to Lgr5+ CBC cells and all differentiated intestinal epithelial cell types.	^41^
Lrig1^a^	Lrig1+ cells are quiescent ISCs that repopulate the damaged crypt following irradiation. Apc loss in Lrig1+ cells generates colonic adenoma.	^42^
Clu	Clu+ cells are extremely rare under normal condition and often appear at position +4. Upon intestinal injury, Clu+ cells expand in a Yap1-dependent manner and reconstitute damaged crypts to regenerate the intestinal structure.	^46^
Progenitors that serve as regenerative stem cells		
Dll1	Dll1+ secretory progenitors can revert to Lgr5+ cells and form long-lived clones along the villus axis, consisting of both absorptive and secretory cells.	^47^
Alpi	Short-lived Alpi+ enterocyte progenitor cells can dedifferentiate and act as reserve stem cells to replenish the loss of Lgr5+ stem cells and play a protective role upon injury.	^49^
Ascl2	Upon ISC ablation, the regenerating cells above position +5 re-express Ascl2 and dedifferentiate into Lgr5+ CBC cells by activating the regenerative transcriptional program, including Interleukin receptor *Il11ra1*.	^45^
Label-retaining cells (LRC)	LRCs in the crypt are usually secretory progenitors, which can dedifferentiate and regain multipotent stem cell features after tissue injury.	^48^

^a^The specificity of these markers has been debated^16^.

## A brief overview of Wnt/β-catenin signaling

Several conserved signaling pathways, including Notch, transforming growth factor-β (TGF-β), epidermal growth factor (EGF), bone morphogenetic protein (BMP), Sonic hedgehog (SHH), and Wnt, operate in the crypt niche. The orchestration of these different pathways determines the proper balance between the proliferation, quiescence, fate decision, and differentiation of intestinal stem cells and their progeny^6^. Active Notch and EGF signaling, for example, are known to maintain an undifferentiated status and control the number of stem cells in the crypt, while BMP signaling drives their differentiation^31,32^. Among these different signals, however, the canonical Wnt/β-catenin pathway plays a pivotal role in regulating intestinal stem cell homeostasis.

**Fig. 2:**
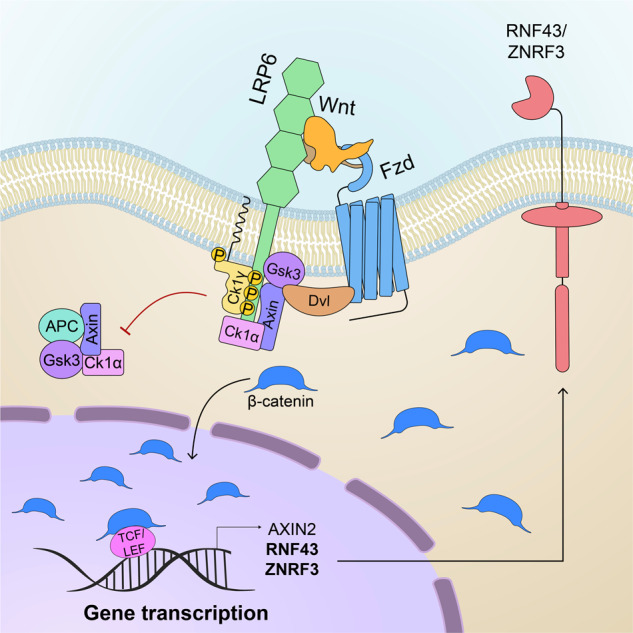
Activation of the canonical Wnt signaling pathway. Binding of Wnt ligands to cognate receptors Frizzled and Lrp5/6 leads to the formation of a multiprotein complex known as the signalosome. The signalosome recruits Dvl, Axin, Gsk3, and casein kinases to the plasma membrane, leading to the dissociation of the cytosolic destruction complex. In turn, this promotes β-catenin stabilization, which translocates into the nucleus. Here, together with TCF/LEF transcription factors, β-catenin induces Wnt target gene activation. Axin2, Rnf43 and Znrf3 are both target genes and inhibitors of the Wnt pathway, thus forming a negative feedback loop.

Wnt ligands belong to a conserved family of secreted glycoproteins modified by palmitoylation on a conserved serine residue^53–56^. Canonical Wnt signaling ultimately depends on the activity of the transcriptional coactivator β-catenin as the main effector protein of the TCF/β-catenin complex. In the absence of Wnt ligands, a cytoplasmic ‘destruction’ complex comprising the tumor suppressors adenomatous polyposis coli (APC), Axin, casein kinase 1 (CK1) and glycogen synthase kinase 3 (Gsk3) binds and promotes the phosphorylation of β-catenin for proteasomal degradation, keeping the Wnt pathway in the OFF state^57^. Conversely, when Wnt ligands bind to their cognate receptors Frizzled (Fzd) and low-density lipoprotein receptor-related protein 5/6 (Lrp5/6), several effectors, including Axin and Disheveled (Dvl), are recruited to the plasma membrane, forming a multiprotein complex known as the signalosome that serves as a platform for downstream effector protein interactions^58,59^. Multiple events, such as the conformational inactivation of Axin, inhibition of β-transducin repeat containing E3 (β-TrCP)-mediated β-catenin ubiquitination and sequestration of negative Wnt regulators into multivesicular endosomes concur to inhibit β-catenin degradation^59–64^. Consequently, stabilized β-catenin shuttles to the nucleus, where it binds the DNA-associated protein T-cell factor/lymphoid enhancer factor (TCF/LEF) and promotes the transcription of downstream Wnt target genes (Fig. 2).

Wnt ligands can transmit signals without β-catenin stabilization, which is denoted as noncanonical Wnt signaling. The noncanonical Wnt pathways are highly diverse, and new branches are continually added. The most well-defined of these pathways are planar cell polarity (PCP) and intracellular calcium signaling, which play critical roles in developmental and physiological processes^65^. Canonical and noncanonical Wnt pathways share several signaling components and retain antagonistic roles to one another, implying the need to maintain a balance between the two^66^. Indeed, recent studies proposed the Wnt/PCP pathway as a new niche signal controlling ISC lineage selection, priming stem cells to adopt Paneth or other secretory cell fates, and intestinal regeneration^67,68^. Understanding the complex network of noncanonical and canonical Wnt pathways could lead to more delicate Wnt signaling manipulation, potentially paving the way for a breakthrough in related disorders.

### Wnt/β-catenin is required for ISC self-renewal and stemness

In the intestine, Wnt/β-catenin activity is highest at the bottom of the crypt, as can be indicated by nuclear β-catenin immunostaining or knock-in Wnt-reporter mouse lines^69^. Previous work suggested that Wnt/β-catenin is fundamental for intestinal crypt formation during the late stages of fetal development. The deletion of TCF-4 (encoded by the *tcf7l2* gene) in mice disrupts the formation of crypt regions with a complete loss of cycling cells from the intervillus regions at Day E18.5, which are instead replaced by differentiated cells^70^. In fact, together with β-catenin, TCF-4 controls a genetic program that promotes the proliferation and undifferentiated status of intestinal cells, in part by upregulating c-Myc (required for cell division) and downregulating p21, which would otherwise induce cell cycle arrest and differentiation^71^. Importantly, TCF-4 maintains a similar role during ISC homeostasis in adult mice, as Cre^ERT2^-mediated conditional deletion in Tcf4^LoxP/LoxP^ mice caused a dramatic reduction in the number of proliferating cells in the crypt as well as in the expression of Wnt targets CD44 and Sox9^72^. *Olfm4*-expressing CBC cells were soon lost after Cre induction, probably because of extensive cell death, followed by a collapse of the crypt-villus structure. Notably, other members of the TCF family, such as TCF-1 or TCF-3/Tcf7l1, were shown to be dispensable in regulating intestinal homeostasis, indicating a specific function for TCF-4^72^. TCF-4 deletion affects the proliferation and survival of not only stem cells but also Paneth cells, which decline rapidly after Cre induction^72^. Comparable to TCF observations, a β-catenin conditional knockout (KO) also resulted in the loss of ISCs and premature differentiation in the adult mouse intestine^73^.

A requirement for secreted Wnt ligands in maintaining ISC homeostasis was indirectly attested by using transgenic mice that overexpressed Dkk1, a well-known extracellular inhibitor specific for canonical Wnt ligands^74,75^. Dkk1 overexpression disrupted intestinal organization and ISC proliferation^76^, similar to TCF-4 or the β-catenin KO. More recently, the importance of Wnt ligands in the intestine has been confirmed using selective chemical inhibitors of Porcupine, an endoplasmic reticulum (ER) membrane-bound O-acyltransferase that is required for Wnt palmitoylation and secretion. Systemic treatments of mice with the Porcupine inhibitors C59 or LGK974 have been shown to greatly downregulate stem cell markers and Wnt target genes and, at high dosages, prevent CBC proliferation and homeostatic self-renewal in the small intestine^77,78^. Several Wnt ligands act redundantly in the crypt to maintain ISC homeostasis^77,79^ and are produced from both epithelial and mesenchymal sources, with Wnt3 being expressed specifically in Paneth cells^80,81^. Using an HA-tagged Wnt3 mouse knock-in line (*Wnt3*^*HA/HA*^), it was possible to visualize the endogenous Wnt ligands in the intestine for the first time. Wnt3 showed minimal diffusion in the crypt, forming a short-range signaling gradient^82^. Interestingly, Wnt3 did not diffuse passively but was instead transferred from Paneth cells (which produce the ligand) to the closest neighboring CBC cells, where it remained tethered to the cell surface through binding to Frizzled. The surface-bound Wnt3 was then disseminated by stem-cell division, generating a gradient of Wnt proteins along the crypt-villus axis^82^.

The power of mouse genetics has been fundamental to further dissecting the role of Wnt signaling components during ISC homeostasis. For instance, Ras-like proto-oncogenes A and B (RalA and RalB), two GTPases involved in Wnt signalosome endocytic activation, were recently shown to be required for CBC maintenance in small intestinal crypts^83^. The conditional KO of the two isoforms, both expressed in the intestinal crypt, resulted in strong reductions in the stem cell markers *Lgr5* and *Olfm4*, with a concomitant decrease in nuclear β-catenin staining in the crypt bottom. Furthermore, single Lgr5^+^ cells isolated from RalA or RalB KO crypts were inefficient in forming organoids, indicating a loss of stemness^83^. In addition to the expression of specific components of the Wnt machinery, other factors, such as physical properties, may contribute to high levels of Wnt signaling. Indeed, a correlation between the compressed cell volume of Lgr5^+^ ISCs and active Wnt/β-catenin signaling has recently been proposed^84^. Among the cellular compartments in the intestinal epithelium, Lgr5^+^ ISCs with greater levels of nuclear β-catenin have a smaller and thinner shape, implying a higher level of physical stress. Interestingly, artificial cell compression enhanced Wnt/β-catenin signaling activity independent of β-catenin release from the cadherin-catenin association, but in a Wnt ligand-dependent manner. Consistently, osmotic and mechanical compression promoted organoid formation from isolated crypts, which often exhibited a cystic, more undifferentiated state, enriching the Lgr5^+^ ISC population and their self-renewal activity^84^. Mechanistically, volumetric compression favors the molecular crowding and stabilization of the Wnt signalosome, which may occur on the basis of higher Wnt responsiveness in ISCs^84^. However, direct in vivo evidence is still missing.

Genetic mutations that cause the unrestrained activation of Wnt/β-catenin signaling induce strong overproliferation and are the cause of many forms of colorectal cancer (CRC). In fact, it is estimated that over 90% of sporadic CRCs contain at least one mutation in a known Wnt regulator^85^. For example, truncating mutations in APC or AXIN2 or mutations that stabilize β-catenin all lead to high levels of nuclear β-catenin/TCF complexes and Wnt activation in intestinal cancers^86–88^. APC alterations are found in 80% of human colorectal tumors, while CTNNB1 (the human homolog encoding β-catenin) and TCF7L2 are mutated only in 5 and 9% of CRCs, respectively^85^. Wnt activating mutations have also been described in regard to other cancers^89^, confirming that Wnt signaling is a major driver in tumor initiation in a variety of organs. APC mutation occurs early in premalignant (benign) lesions of the intestine, such as small polyps^90^, and APC^min^ (multiple intestinal neoplasia) mice carrying a dominant mutation in APC are predisposed to develop multiple intestinal adenomas^91,92^, similar to mutations causing familial adenomatous polyposis (FAP) in humans. The progression from benign adenoma to malignant carcinoma requires the sequential acquisition of additional specific genetic alterations^93^, an event that can be modeled using CRISPR/Cas9 genetically modified intestinal organoids^94,95^. APC mutations are commonly found to activate Wnt/β-catenin signaling and initiate the formation of benign polyps, while the progression to CRC requires activating mutations in the EGF pathway and inactivating mutations in P53 and TGF-β. However, the restoration of APC is sufficient to sustain cell differentiation, homeostatic proliferation, and tumor regression in CRC with multiple genetic mutations^96^, suggesting that even advanced cancers are still dependent on the constitutive activation of Wnt. Altogether, these data indicate that Wnt/β-catenin signaling is required for ISC proliferation and maintenance, while its abnormal activation is a major cause of tumor formation.

## Rnf43 and Znrf3 are fundamental feedback inhibitors of Wnt signaling in the intestinal crypt

**Fig. 3:**
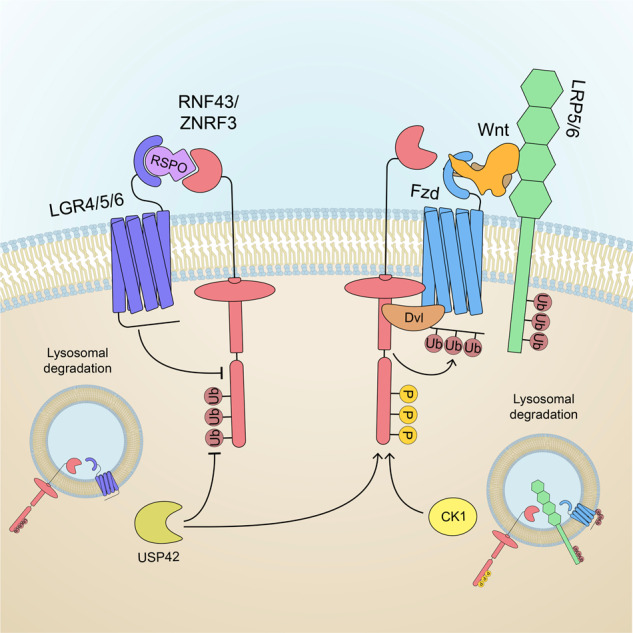
Rnf43 and Znrf3 are transmembrane E3 ligases that promote Wnt receptor turnover. Rnf43/Znrf3 (R/Z) promotes Frizzled and Lrp5/6 ubiquitination, assisted by the cytosolic adaptor Dvl. Once ubiquitinated, Wnt receptors are internalized by endocytosis and degraded via the lysosomal system. R/Z activity is regulated by the phosphorylation of the cytosolic tail by casein kinase 1 (CK1). The secreted Wnt agonist Rspo inhibits R/Z by forming a ternary complex with Lgr4/5/6 receptors, which in turn triggers the autoubiquitination of R/Z followed by the endocytosis of the complex. The deubiquitinase Usp42 stabilizes R/Z at the plasma membrane by removing ubiquitin from R/Z proteins, hence inhibiting Wnt signaling.

Due to its strong mitogenic activity, several mechanisms have evolved to maintain Wnt/β-catenin signaling under tight control. Multiple inhibitors operating at different levels of the Wnt/β-catenin signaling cascade, including Axin^97^ and SH3 domain-binding protein 4 (SH3BP4)^98^, contribute to maintaining intestinal homeostasis. However, it seems that the main feedback mechanism ensuring adequate levels of Wnt signaling in the intestinal crypt occurs at the plasma membrane. In 2012, a screening of Wnt target genes expressed in the intestine revealed that two closely related single-pass transmembrane E3 ubiquitin ligases, Rnf43 and Znrf3 (R/Z), are specifically expressed in crypt stem cells in response to Wnt/β-catenin signaling^17^. Importantly, it was shown that R/Z dampens Wnt signaling through the ubiquitination of Frizzled and Lrp5/6 receptors, which are then degraded through the endolysosomal pathway (Fig. 3)^17,99^. Of note, Dvl is required for the R/Z-mediated degradation of Wnt receptors^100^. Hence, R/Z acts as a Wnt feedback inhibitor at the receptor level. The KO of these genes in the mouse intestine elicited the dramatic proliferation and expansion of crypt cells, with the formation of adenomas similar to the loss of APC, thus revealing their tumor suppressor nature^17^. Nonetheless, unlike APC mutations, R/Z-deficient cells still depend on a source of paracrine Wnt ligand^101^.

Notably, R/Z is the target of stem cell-specific Lgr receptors, and several studies have contributed to elucidating the underlying mechanistic regulation between R/Z and Lgr. Lgr4/5/6 are members of a subfamily of G-protein coupled receptors, characterized by a large extracellular domain containing 16–18 leucine-rich repeats and expressed in several types of stem cells^102^. Importantly, the Lgr leucin-rich domain interacts with R-spondins, a long-known class of 4 secreted Wnt agonists (Rspo 1–4)^103–107^. When Rspo binds to Lgr4/5/6, they promote the formation of a trimeric complex containing R/Z, followed by clearance of the E3 ligases from the plasma membrane as a result of autoubiquitination (Fig. 3)^99,108^. In this way, the Wnt receptors become stabilized, and cells are sensitized (i.e., respond more robustly) to Wnt ligands. Accordingly, Rspo/Lgr have no signaling activity per se but sustain a robust Wnt signal. Together with R/Z, both Lgr and Rspo play a fundamental role in regulating stem cell activity in the intestinal crypt through the modulation of Wnt signaling. The conditional KO of Lgr5 and its close homolog Lgr4 caused a rapid loss of stem cells and a strong reduction in CBC marker expression^107^. Conversely, the overexpression of Rspo is known to induce the strong proliferation and expansion of the stem cell compartment in the small intestine, leading to the formation of adenomas in a similar way to R/Z depletion^109–111^. Since Wnt ligand diffusion in the crypt requires tethering on stem cell membranes through Fzd, the interplay between R/Z and the Rspo/Lgr signaling system plays an active role in determining the shape and size of the Wnt signaling gradient through the modulation of Wnt receptor turnover^82^. Unsurprisingly, RNF43 mutations or RSPO2/3 chromosomal translocations, both of which boost Wnt signaling, are associated with different forms of colorectal tumors (with mutation frequencies of 18 and 3–8%, respectively^108,112^), as well as pancreatic and gastric cancers^112–120^. These mutations are mutually exclusive with APC mutations^112,117^, and in contrast to the latter, they are all characterized by their dependency on a source of Wnt ligand (Wnt addiction). Thus, Rspo/Lgr/RZ are prominent players in regulating the homeostasis of ISCs as well as other tissue-specific adult stem cells^102^.

Until recently, it has remained unclear how posttranslational modifications regulate R/Z^121^. CK1-mediated phosphorylation in the serine-rich motif near the Dvl interacting region (DIR) on the C-terminal tail of Rnf43 acts as a “phospho-switch” to increase its Wnt-antagonistic activity^122^. This region is often mutated or truncated in oncogenic forms of Rnf43, which consequently eliminates the negative regulation of Fzd^122^. CK1 phosphorylation may also support an alternative Wnt-inhibitory function of Rnf43, requiring interactions with the Wnt destruction complex independent of Fzd turnover^123^. Certain cancer-associated Rnf43 truncated mutants promote Wnt signaling while still retaining the ability to downregulate Fzd. This is due to a stronger association with Axin and CK1, which trap them at the plasma membrane and cause the dissociation of the destruction complex, activating the Wnt pathway at the level of β-catenin-mediated transcription^123^. On the other hand, dephosphorylation by the tumor suppressor Ptprk is essential for Znrf3 to promote Fzd downregulation in an endocytosis-dependent fashion^124^. Thus, it is possible that phosphorylation differentially regulates the two functional homolog E3 ligases.

Finally, it is well established that Lgr regulates R/Z clearance through autoubiquitination^99,108^, thereby promoting Wnt receptor stabilization. Interestingly, R/Z autoubiquitination is also a dynamic process and is reversed by Usp42, a deubiquitinase that stabilizes R/Z levels at the plasma membrane, counteracting Rspo/Lgr4-mediated clearance and promoting Wnt inhibition^125,126^. Usp42 mutations are found in colorectal cancer, and Usp42 KO organoids can grow in the absence of Rspo^125^, similar to R/Z double KO organoids^101^. The complexity of R/Z modulation has only begun to be unraveled, and the identification of novel factors and mechanisms involved in the E3 ligase regulatory network highlights the importance of these Wnt antagonists in stem cell and cancer biology.

## ISCs are regulated by neutral drift dynamics

**Fig. 4:**
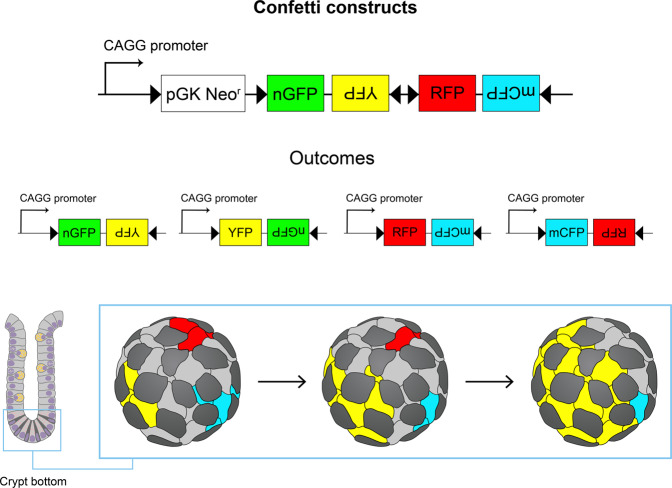
Confetti reporter-based multicolor lineage tracing can be used to study neutral drift dynamics in stem cell populations. Top: the *R26R-confetti* allele functions as a stochastic multicolor reporter, in which Cre-dependent recombination induces the mutually exclusive expression of one of four different fluorescent proteins (green, yellow, red, and cyan). The specific activation of confetti in intestinal stem cells allows us to follow clonal dynamics in the intestinal crypt, as individual ISCs and their direct progeny express the same unique fluorescent protein. Bottom: an intestinal crypt (visualized from a bottom view) containing ISCs labeled with different fluorescent proteins (red, yellow, and cyan) or unlabeled (light gray). Paneth cells (dark gray) are intercalated between the ISCs. Over time, some clones will expand (see the yellow clone in the example), while others will shrink or disappear completely (cyan and red clones, respectively) as a result of neutral competition.

A long-standing question in the stem cell field is how ISCs are maintained and how the choice between different fates is regulated. A popular model from studies on *Drosophila* germline stem cells and *C. elegans* proposes that the asymmetric division of stem cells generates two daughter cells with divergent fates: a new stem cell and a TA progenitor that will differentiate^127,128^. It has been argued whether ISC homeostasis is also governed by asymmetric division. However, there is no evidence that CBC cells adopt unequal fates based on intrinsic mechanisms. Rather, it seems that the choice between self-renewal and differentiation is stochastic. Elegant lineage-tracing experiments using mice expressing a multicolor fluorescent reporter (denoted as ‘confetti’) (Fig. 4), together with quantitative mathematical models, allowed the analysis of CBC stem cell dynamics^129,130^. Stem cell clones (expressing one of four different fluorescent proteins) either randomly expanded to eventually colonize the entire crypt or were lost and replaced by other clones (expressing a different fluorescent protein). Surviving clones maintained the pool of Lgr5^+^ stem cells, while clones comprising only cells undergoing differentiation soon became extinct, losing their fluorescent trace (Fig. 4). By 2 months after the start of lineage tracing, almost all the crypts had become monoclonal (or fixed, that is, each crypt derived from a single stem cell expressing one particular fluorescent protein). Therefore, CBC stem cells are all endowed with the same long-term stemness potential and compete equally (hence, the term ‘neutral competition’) for the available niche space, the fundamental determinant for their ultimate fate. Upon cell division, cells that are pushed out of the niche space will undergo differentiation, while those that remain at the base will maintain their self-renewal capability^131^. In other words, cells at the base of the crypt have a higher chance of surviving and forming clones that will repopulate the entire crypt, while those in the upper part of the crypt are more likely to be pushed into the TA zone, after which they will undergo differentiation. Thus, intestinal stem cell homeostasis is maintained at the population level, where the loss of stem cells is compensated by the generation of other stem cells. Over time, one stem cell clone will eventually outcompete the others by neutral drift dynamics, substantiating the long-known notion that crypts tend to become monoclonal (Fig. 4)^132,133^.

What factors limit the available niche space? A possible answer is contact with Paneth cells, which produce many of the growth factors required for stem cell maintenance. The genetic depletion of Paneth cells in Sox9^fl/fl^ mice results in stem cell loss, and coculturing Paneth cells with isolated Lgr5^+^ cells maximizes organoid formation^81^. Paneth cells intermingle with CBC cells (which are squeezed in between) and organize together into an icosidodecahedron geometry, similar to the tessellation of a soccer ball (Fig. 4). This spatial arrangement maximizes the contact between stem cells and Paneth cell membranes, to which some of the signaling factors required for stem cell renewal are bound, such as Wnt3^82^. Thus, Paneth cells are likely the prime controllers for establishing the ISC niche by producing and possibly confining growth factors.

In addition to Paneth cells, several studies have shown that mesenchymal cells in the stromal niche underlying crypts or the villus tip also play a role in intestinal stem cell maintenance. Different subgroups of rare mesenchymal cells have been identified, such as Gli1-expressing cells^134^, Foxl1^+^ telocytes^135^, Pdgf receptor-α (PdgfRα)^+^ pericryptal myofibroblasts^136^, Lgr5^+^ telocytes^137^, Ng2^+^ pericryptal cells^138^, Gremlin1^+^ trophocytes^139^ and Map3k2-regulated stromal cells^140^. Altogether, these cells provide secreted factors, such as Wnts and R-spondins, indispensable for ISC growth and maintenance in vivo during homeostasis and regeneration and can support Lgr5-derived organoid growth when cocultured in vitro.

Although the complete organization of the ISC niche is still elusive, these data point to a model where Lgr5^+^ CBC cells compete for stemness factors that are spatially restricted in the confined space of the crypt niche, and the R/Z-Rspo-Lgr axis plays a prominent role in regulating the stem cell response to these factors. Interestingly, similar competition mechanisms may operate in the stem cell compartments of other tissues with sustained turnover, such as the gastric tissue^141,142^ or the skin^143^, potentially working as a general mechanism underlying adult stem cell homeostasis.

## Winning the clone wars: Cancer cells induce healthy neighbors to join the dark side

It is still incompletely understood how cell competition dynamics work in the context of diseases such as cancer. Following neutral drift dynamics, ISCs can randomly be replaced by any other equipotent ISC present in the crypt. This also means that an ISC that has acquired a specific mutation also has a chance of being replaced by other wild-type (WT) cells. Cell competition is an evolutionarily conserved process that prevents the accumulation of harmful mutations and eliminates unfit cells and thus plays an important role in maintaining tissue health^143–145^. However, what happens when a mutation confers a fitness advantage over neighboring normal cells, as is the case for many oncogenic mutations? The loss of APC in ISCs leads rapidly to the formation of intestinal adenomas in mice^146^. Mutated ISCs are more efficient in generating tumors than progenitor or differentiated cells, which have a short lifespan and are usually shed off into the intestinal lumen within 3–4 days^146^. As revealed by previous studies, precancerous ISCs with a Kras^G12D^ activating mutation or APC^−/−^ show a substantial survival advantage over normal stem cells, with a higher probability of crypt fixation due to biased drift^147,148^. The expansion of mutated cells predisposes a tissue to cancer development and progression, a process known as field cancerization. Nonetheless, the presence of such advantageous genetic alterations does not guarantee immediate success in colonization, as only a relatively low number of APC^−/−^ or Kras^G12D^ stem cells will finally reach crypt fixation^147^. In fact, most mutated cells can still be eliminated stochastically by surrounding normal cells through competition mechanisms.

**Fig. 5:**
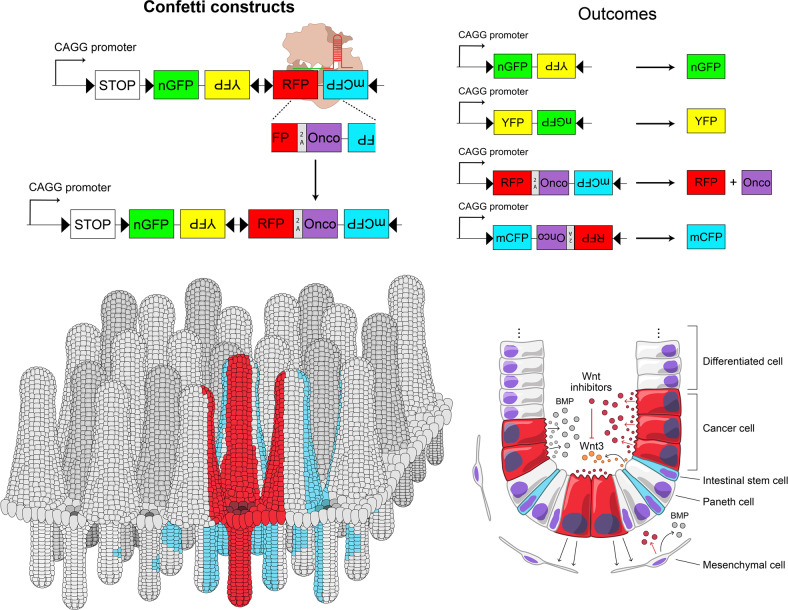
Red2Onco system allows us to study the effects of cancer cells on their surrounding environment. Top: Red2Onco design is based on the original *confetti* allele. CRISPR/Cas9 genome editing was used to insert a 2 A peptide-Oncogene cassette between the red and cyan fluorescent proteins. This converts the original RFP into a bicistronic construct coexpressing the red fluorescent marker together with an oncogene of choice. Cre recombination occurs as usual, inducing the stochastic expression of one of the four fluorescent reporters. Cells expressing RFP will also express the selected oncogene, while the other colors label wild-type (WT) cells. Bottom: expression of an oncogene, such as *Kras*^*G12D*^ or *PIK3CA*^*H1047R*^, confers a survival advantage to red clones, which will rapidly expand into their home crypt. Over time, this will lead to the fixation of the mutant clone into the crypt, which becomes monoclonal red. Due to the cancer cell influence, nearby crypts will also accelerate fixation and become monoclonal (visualized in cyan). Distal crypts are not affected by the red mutant clones. Mutant cells express secreted factors, including prodifferentiation signaling molecules (BMPs) and Wnt antagonists (Notum, Dkk, and Sfrp), which reduce the number of stem cells in proximal WT crypts, driving biased drift. Note that niche remodeling induced by cancer cells also affects nearby stromal cells.

What allows genetically altered cells to eventually subjugate normal, healthy cells and win the competition? To investigate this, our lab has recently used a multicolor reporter system denoted as Red2Onco, derived from the original confetti allele, in which the expression of a red fluorescent protein (RFP) was tied to the overexpression of a specific oncogene (Fig. 5)^149^. This approach makes it possible to trace clones derived from mutant and WT cells in the same tissue, such as the intestine. Mutations leading to the constitutive activation of the Ras and phosphoinositide 3-kinase (PI3K)-AKT pathways are frequently found in several malignancies, including CRC^85,150^. Interestingly, we found that ISC clones overexpressing *Red2Onco Kras*^*G12D*^ or *PIK3CA*^*H1047R*^ showed a substantial survival advantage over WT cells, which rapidly led to the fixation of monoclonal red crypts in the small intestine, as expected by biased competition dynamics. Rather unexpected, however, was the finding that mutant crypts could accelerate clonal fixation in proximal, adjacent WT crypts (labeled with either yellow, blue, or green fluorescent proteins)^149^. This correlated with a decrease in the number of Lgr5^+^ WT stem cells and an increase in differentiation markers. A comparative transcriptomic analysis of FACS-sorted mutant and WT cells revealed that our Red2Onco models showed a marked increase in BMP signaling due to the higher production of Bmp2/7 from mutant cells, providing an explanation for the loss of stemness and increase in differentiation. Notably, stromal cells from the mesenchymal population also showed an increase in the expression of the Wnt antagonists secreted Frizzled-related protein 2 and 4 (Sfrp2 and 4). This suggests that the oncogene-driven remodeling of the niche environment renders WT cells unfit to compete, favoring the fixation of mutant cells in the intestinal crypts^149^.

Two additional works have shown similar findings using a different experimental approach. Intestinal organoids derived from APC^−/−^ exhibited growth and survival advantages and rapidly overcame WT organoids in coculture experiments^151,152^. Of note, APC^−^^/−^ not only passively outcompeted WT organoids but also actively reduced their clonogenic and expansion capacity. Importantly, the same effect was observed when WT organoids were grown in conditioned medium (CM) derived from mutant cells, clearly indicating a noncell autonomous effect. A transcriptomic profile analysis of APC^−^^/−^ cells revealed an increase in the expression of several extracellular Wnt inhibitors, such as Wnt inhibitory factor (Wif), Dkk, Sfrp, and Notum^151,152^, as also shown by in situ hybridizations on APC^−^^/−^ tumors in vivo. Among the Wnt antagonists, Notum seemed more effective in inhibiting ISC-derived organoid growth and expansion in vitro^152^, although a cooperative effect with the other antagonists is likely to occur^151^. Notum is a secreted deacylase that removes palmitoleic acid from Wnts, disengages them from Fzd, and suppresses Wnt signaling^153,154^. Similar to what was reported by Yum et al.^149^, Wnt inhibition reduced the expression of transcripts associated with CBC stemness, while the expression of differentiation genes increased^151,152^. Notably, APC KO cells are shielded from Notum activity, as Notum acts at the ligand–receptor level, while APC loss activates the Wnt pathway downstream of receptor signaling. It was reported that the prevention of Wnt secretion by Porcupine inhibitors favors tumor initiation by reducing the pool of functional stem cells^78^. Thus, a common scheme emerges from these recent studies, where cancer cells actively reduce the competitive fitness of neighboring WT stem cells by promoting their differentiation, with the consequent loss of their stemness potential. Mutated cells achieve this by utilizing paracrine signaling, e.g., by secreting growth factor inhibitors (e.g., Notum or other Wnt inhibitors) or prodifferentiation factors (such as BMPs), while themselves are “protected” or “surviving” from this suppressive activity^149,151,152^ (Fig. 5). Rescue experiments further corroborated the specificity of Wnt inhibition in biased competition: the use of Notum chemical inhibitors, Notum genetic knockout, or treatments with the Gsk3 inhibitor LiCl (which activates β-catenin-dependent transcription) were all effective in reducing crypt fixation by APC^−/−^ cells by increasing the number and competitive fitness of WT ISCs.

Interestingly, Notum plays a similar role in the developing wing disc of *Drosophila melanogaster*: cells with increased Wnt activity (as a result of APC or Axin mutations) eliminate surrounding cells by triggering apoptosis^155^. The supercompetition of APC or Axin mutant cells is prevented by genetic mutations inactivating Notum, hence suggesting that the secreted deacylase has an evolutionarily conserved role in Wnt-dependent competition. Finally, the effects of Notum on ISCs are known in the context of the aged intestine. In old Paneth cells, the mammalian target of the rapamycin complex 1 (mTORC1) pathway produces higher levels of Notum, which, in turn, reduces Wnt activity and stem cell numbers, thus limiting the regenerative ability of the aged intestine in response to injury^156^. This finding provides a possible mechanistic link between age and the increased risk of CRC.

## Conclusions

Most studies on cancer biology have focused on the cell-autonomous effects that genetic alterations have on the same cells carrying these mutations. However, it is now evident that cancer cells actively modify their surrounding environment for their own benefit, altering the homeostatic cell competition dynamics to render WT cells as weak competitors. Biased drift, as opposed to neutral competition, accelerates the fixation of certain mutations, from which more aggressive, malignant cancer evolves. Importantly, these changes in cell competition occur through the dysregulation of the same signaling pathways responsible for stem cell maintenance, such as Wnt/β-catenin. Given that Wnt-driven cancers often express high levels of Wnt inhibitors as a negative feedback program, recent discoveries highlighting this expression as a critical mechanism for cancer supercompetition may open up new strategies for pharmacological treatment. Instead of aiming to inhibit Wnt signaling, a difficult task to achieve in APC^−/−^ tumors, it may be more efficient to interfere directly with cancer-derived Wnt antagonists, or vice versa, to increase Wnt activity in WT stem cells, improving their competition capacity. Thus, further studies are needed to address how tumors with different genetic backgrounds modify the niche environment to outcompete the surrounding normal cells.
